# Identification of Individuals at Increased Risk for Pancreatic Cancer in a Community-Based Cohort of Patients With Suspected Chronic Pancreatitis

**DOI:** 10.14309/ctg.0000000000000147

**Published:** 2020-04-04

**Authors:** Christie Y. Jeon, Qiaoling Chen, Wei Yu, Elizabeth Y. Dong, Joanie Chung, Stephen J. Pandol, Dhiraj Yadav, Darwin L. Conwell, Bechien U. Wu

**Affiliations:** 1 Samuel Oschin Cancer Institute, Cedars-Sinai Medical Center, Los Angeles, California, USA;; 2 Department of Epidemiology, UCLA Fielding School of Public Health, Los Angeles, California, USA;; 3 Research and Evaluation, Kaiser Permanente Southern California, Pasadena, California, USA;; 4 Center for Pancreatic Care, Kaiser Permanente Los Angeles Medical Center, Los Angeles, California, USA;; 5 Division of Digestive and Liver Diseases, Cedars-Sinai Medical Center, Los Angeles, California, USA;; 6 Division of Gastroenterology, Hepatology and Nutrition, University of Pittsburgh Medical Center, Pittsburgh, Pennsylvania, USA;; 7 Division of Gastroenterology, Ohio State University Wexner Medical Center, Columbus, Ohio, USA.

## Abstract

We lack reliable methods for identifying patients with chronic pancreatitis (CP) at increased risk for pancreatic cancer. We aimed to identify radiographic parameters associated with pancreatic cancer in this population.

**METHODS:**

We conducted a retrospective cohort study of patients with suspected CP within an integrated healthcare system in Southern California in 2006–2015. Patients were identified by a diagnostic code and confirmed by imaging findings (parenchymal calcification, ductal stones, glandular atrophy, pseudocyst, main duct dilatation, duct irregularity, abnormal side branch, or stricture) defined by the natural language processing of radiographic reports. We used Cox regression to determine the relationship of smoking, alcohol use, acute pancreatitis, diabetes, body mass index, and imaging features with the risk of incident pancreatic cancer at least 1 year after abnormal pancreas imaging.

**RESULTS:**

We identified 1,766 patients with a diagnostic code and an imaging feature for CP with a median follow-up of 4.5 years. There were 46 incident pancreatic cancer cases. Factors that predicted incident pancreatic cancer after 1-year of follow-up included obesity (hazard ratio 2.7, 95% confidence interval: 1.2–6.1) and duct dilatation (hazard ratio 10.5, 95% confidence limit: 4.0–27). Five-year incidence of pancreatic cancer in this population with duct dilatation was 6.3%.

**DISCUSSION:**

High incidence of pancreatic cancer in suspected patients with CP with pancreatic duct dilatation warrants regular surveillance for pancreatic cancer.

## INTRODUCTION

Chronic pancreatitis (CP) is an established risk factor for pancreatic cancer (1–3), a leading cause of cancer-related mortality among patients with CP (4). However, routine screening for pancreatic cancer in this population is not recommended based on the relatively low absolute risk of cancer and potential harms associated with screening (5). Therefore, improved methods for early cancer detection in patients with CP are a key step to improve the outcome in this patient population.

Retrospective analyses of the real-world clinical data have identified an excess risk of pancreatic cancer soon after a diagnosis of CP (6), raising concern that early cancers may be misdiagnosed as CP. Apart from the risk of missing an occult neoplasm, previous literature has also consistently identified higher long-term risk of pancreatic cancer among patients with CP than in the general population (1–3). To date, it remains unclear which patients with CP are at increased risk for developing pancreatic cancer in the future. Addressing this gap in knowledge is an important step to develop effective strategies for the early detection of cancer in these patients.

The objective of this study was to assess clinical and radiographic features associated with increased risk of pancreatic cancer after imaging for CP diagnosis. Considering that the cost effectiveness of future diagnostic or screening recommendations depends on the probability of discovering pancreatic cancer, we also aimed to estimate the age- and sex-specific incidence rates of pancreatic cancer in the CP population and the relative incidence as compared to the general population.

## METHODS

### Study design and setting

We conducted a retrospective cohort study of patients diagnosed with CP within Kaiser Permanente Southern California (KPSC) between January 2006 and December 2015. Kaiser Permanente is a community-based integrated healthcare system comprising 7 distinct regions, of which Southern California is one of the largest. The present study was approved by the Institutional Review Board of KPSC and the Consortium for the Study of Chronic Pancreatitis, Diabetes, and Pancreatic Cancer.

### Patient population

Patients with CP were identified through diagnosis codes (International Classification of Disease [ICD]-9 577.1) with additional confirmation by the presence of at least 1 radiographic feature suggestive of CP on computed tomography, transabdominal ultrasound, or magnetic resonance imaging (MRI). When patients had multiple imaging procedures performed, we selected the earliest image demonstrating an abnormal finding as the date of cohort entry. Among images conducted on patients with suspected CP, the most common radiographic studies were computed tomography (52%), followed by transabdominal ultrasound (23%) and MRI (9%). Patients with a history of pancreatic cancer before the first radiographic image with a positive feature for CP, those with imaging performed before 2006, those with less than 1 year of continuous membership before the imaging procedure, and those with less than 1 year of follow-up after an abnormal pancreas radiograph were excluded. Occult, prevalent pancreatic cancer cases were excluded, along with persons with follow-up less than 1 year.

### CP imaging feature set (natural language processing)

One of the key challenges in characterizing imaging features among patients with CP is the lack of structured codes or established template for defining specific radiographic features. To address this limitation, we developed a natural language processing (NLP) algorithm to identify the imaging features reported on the free text of findings and impressions focused on the pancreas in cross-sectional imaging studies. We developed search strings for the following radiographic features of CP based on the established parameters from clinical guidelines (7): atrophy, calcification, pseudocyst, main duct dilatation, pancreatic duct irregularity, intraductal stone/calculus, intraductal filling defect, abnormal side-branch, and pancreatic duct stricture.

We used an iterative process for each of the factors combining chart validation with algorithm refinement until >98% accuracy was achieved compared with a manual chart review for 300 patients with CP. An in-house NLP software platform was used to develop a series of imaging algorithms. The software package was developed based on an open-source Application Programming Interface including Natural Language Toolkit, NegEx/pyConText, and Stanford CoreNLP (8–10). The NLP algorithm for the CP image was developed in sequential steps. First, the imaging report was preprocessed for section and sentence boundary detection. Second, we used text patterns to map and classify analogous terms. Third, the distance between key words, such as “pancreatic duct” and “irregular,” were measured and algorithms were developed to apply maximum distance between words. Fourth, the absence of features was detected through the negation algorithm pyContText/NegEx. Fifth, features described outside of certain temporal boundaries (e.g., historical finding) were detected by pyConText and excluded. Finally, the NLP note-level results were combined into patient-level results.

### Validation of CP

Relying on the ICD codes alone to identify CP has suboptimal positive predictive value (11), we therefore required an abnormal pancreas imaging result in addition to ICD codes to identify patients with CP. Although persons with calcification or calculi most definitively have CP, other features are less definitive for CP. We therefore performed a manual review of electronic health records and imaging reports of 10% of patients with CP without calculi or calcification. The reviewer was blinded to the NLP-discovered features and the eventual pancreatic cancer status of the patient.

### Outcome assessment

Pancreatic cancer cases included patients with at least 2 outpatient or inpatient visits with the diagnosis codes (ICD-9 157.*), patients registered in the internal KPSC cancer registry as having a malignant neoplasm in the pancreas and those with pancreatic cancer as cause of death in the Death Index. All cancer cases were confirmed through the manual chart review, and only primary pancreatic cancer cases were included. The date of diagnosis was defined as earliest of the following: date of the healthcare visit with pancreatic cancer–specific ICD-9 code, the date of pancreatic cancer diagnosis in the tumor registry, or the date of death due to pancreatic cancer. Time-to-event was defined as days from 12 months of the first positive pancreas imaging to pancreatic cancer diagnosis. Patients were censored by death due to causes other than pancreatic cancer, discontinuation of KPSC membership, or December 31, 2016, whichever came first.

### Covariate definitions

Age was determined at the time of the first physician encounter with a diagnosis of CP. We also extracted data on sex and race, classified as non-Hispanic white, non-Hispanic black, Asian, Hispanic and others. Current consumption of alcohol (yes vs no) and smoking status (current, former, or never) were determined by self-report based on the most updated health status data available on or before cohort entry. Body mass index (BMI) was assessed at the time of the abnormal imaging result. History of acute pancreatitis and diabetes *before cohort entry* was assessed by ICD-9 codes 577.0, 250.xx, respectively.

### Data analysis

We first constructed a cumulative incidence plot of pancreatic cancer using the Kaplan–Meier survival method. The demographic and clinical characteristics of the CP population were described by the pancreatic cancer status. We then used Cox regression with Firth penalized partial likelihood approach to evaluate the risk factors associated with the development of pancreatic cancer, given the limited number of events. Covariates in the model included patient-related factors (age, sex, race/ethnicity, alcohol consumption, smoking, diabetes, BMI, and previous history of acute pancreatitis) and radiographic features (atrophy, calcification, pseudocyst, main duct dilatation, pancreatic duct irregularity, intraductal stone/calculus, intraductal filling defect, abnormal side-branch, and pancreatic duct stricture). We additionally performed a competing risk analysis by Fine and Gray regression with death due to causes other than pancreatic cancer as a competing risk to confirm that covariate-pancreatic cancer outcomes were not influenced by mortality unrelated to pancreatic cancer.

In addition, to estimate the age- and sex-specific incidence rates of pancreatic cancer, we determined the number of incident pancreatic cancer by age groups (≤40, 41–50, 51–60, 61–70, 71–80, and ≥81) and sex in the overall cohort and divided by the person-years observed. The age- and sex-specific incidence rates of pancreatic cancer in the non-CP population were also estimated for comparison and the age- and sex-standardized incidence rate ratios of pancreatic cancer were computed. Statistical analysis was performed with SAS statistical software, version 9.4 (Cary, NC), and all reported *P* values are 2-sided with alpha 0.05.

## RESULTS

**Figure 1.**
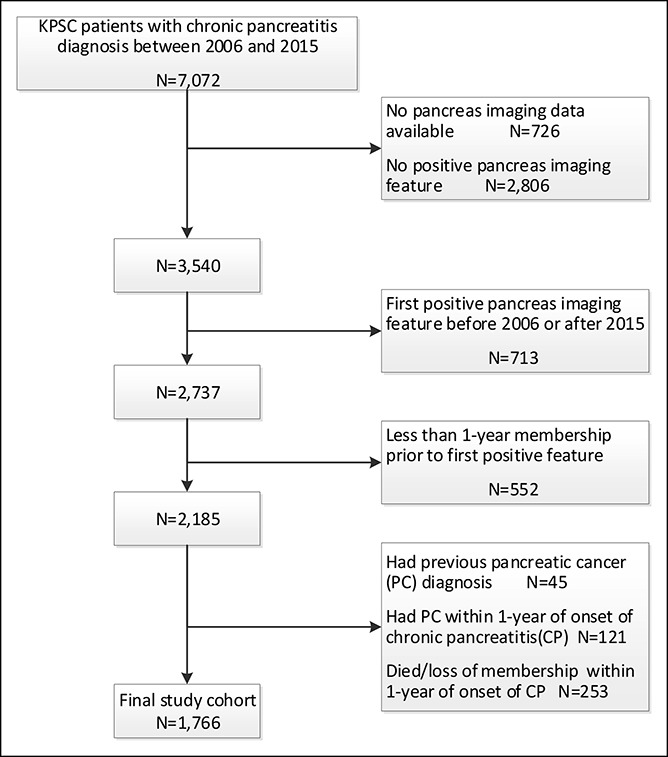
Selection of patients with suspected chronic pancreatitis with abnormal radiographic pancreas imaging. KPSC, Kaiser Permanente Southern California.

After applying our inclusion and exclusion criteria, we identified a total of 1,766 patients diagnosed with CP who had at least 1 abnormal pancreatic finding on radiographic imaging and who had survived at least 1 year without pancreatic cancer (Figure 1). Of 86 patients with coded diagnosis of CP without calcification or calculi at first positive imaging who were subject to the manual chart review, 36 (42%) had or developed definitive CP based on the endoscopic ultrasound findings or based on the Cambridge 3 or 4 classification on MRI/computed tomography imaging. Another 43 (50%) had or developed acute pancreatitis without definitive CP, of whom 20 (23%) were documented to have severe acute pancreatitis. Seven (8%) had no clear evidence of CP or acute pancreatitis, whereas one of these patients had a pancreatic pseudocyst and another had elevated CA19-9.

A descriptive summary of the study cohort is presented in Table 1. The median duration of follow-up after positive pancreas imaging was 4.5 years (interquartile range: 2.5, 7.0 years). The mean age at cohort entry was 60.1 years (SD 16.0), and 46% of the cohort were women. Thirty percent of the population reported regularly consuming alcohol at cohort entry. Twenty percent of the patients in the study cohort were current smokers, with an additional 33% having been a smoker in the past. Overall, 48% of patients had a history of acute pancreatitis and 44% of patients had a pre-existing diagnosis of diabetes before being diagnosed with CP. A few patients (n = 10, 0.6%) had a diagnosis of intraductal papillary mucinous neoplasm (IPMN) at the time of imaging procedure. The median time to incident pancreatic cancer was 2.4 years.

**Table 1.**
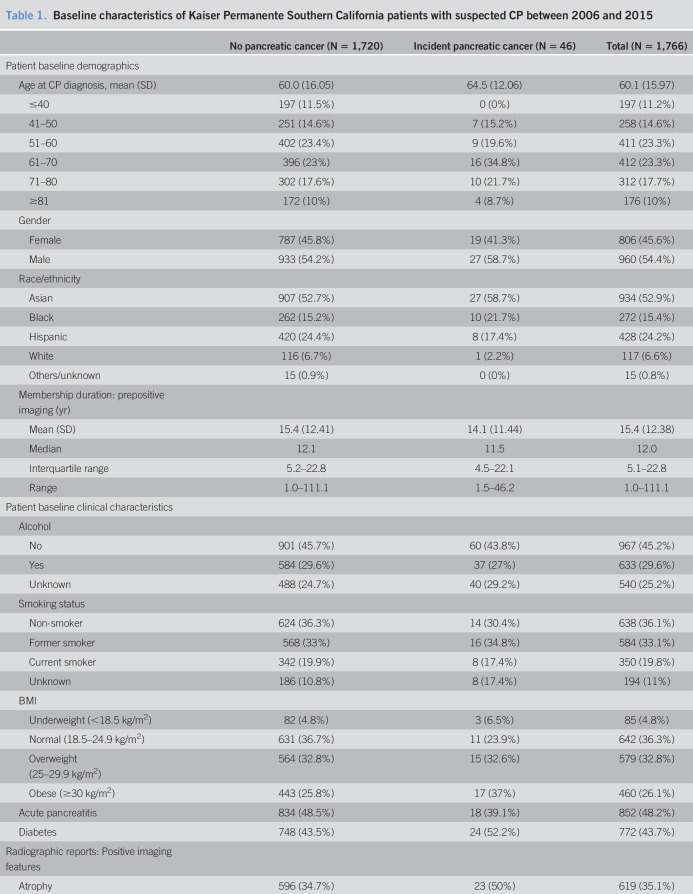

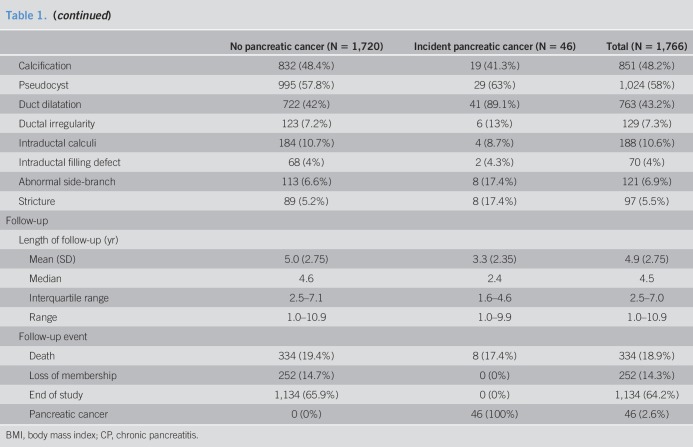
Baseline characteristics of Kaiser Permanente Southern California patients with suspected CP between 2006 and 2015

Of the CP imaging features, pseudocyst was the most common abnormality (58%) followed closely by parenchymal calcification (48%). Ductal dilation was present in 43% of the total study population and present in most patients who went on to be diagnosed pancreatic cancer (89%).

**Figure 2.**
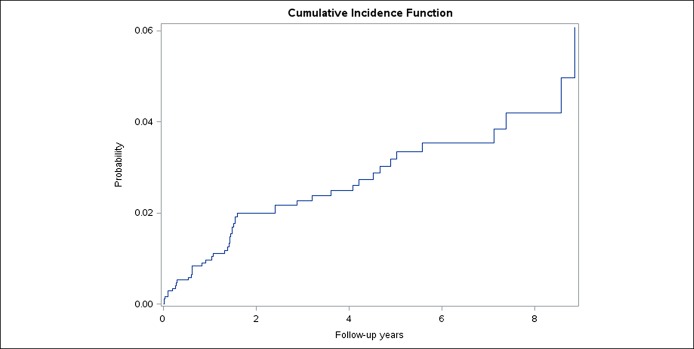
Cumulative incidence curve of pancreatic cancer ≥1 year after abnormal pancreas imaging in patients with suspected chronic pancreatitis.

A total of 46 patients developed pancreatic cancer during the study period, 2 of whom had IPMN at baseline. An incidence plot for pancreatic cancer since 1 year after abnormal pancreas imaging in suspected patients with CP is presented in Figure 2. Incidence of pancreatic cancer increased steadily, and estimated cumulative incidence of pancreatic cancer was 2% after 2 years of follow-up and was 3.2% after 5 years of follow-up.

Table 2 presents the results of Cox proportional hazards regression for incident pancreatic cancer. Although age was associated with increased risk of pancreatic cancer, other patient-related factors such as pre-existing diabetes, smoking, or alcohol history were not associated with increased risk of pancreatic cancer after adjusting for findings on radiographic imaging. Being obese (hazard ratio [HR] 2.7, 95% confidence limit [CL]: 1.2–6.1) and duct dilatation (HR 10.5, 95% CL: 4.0–26.1) were independently associated with increased risk of pancreatic cancer. Of note, being underweight was also marginally associated with pancreatic cancer (HR 3.1, 95% CL: 0.9–10.8), demonstrating that the association of BMI with pancreatic cancer is not monotonic. A competing risk analysis by Fine and Gray regression confirmed that our findings on the factors associated with pancreatic cancer were not influenced by death due to causes other than pancreatic cancer.

**Table 2.**
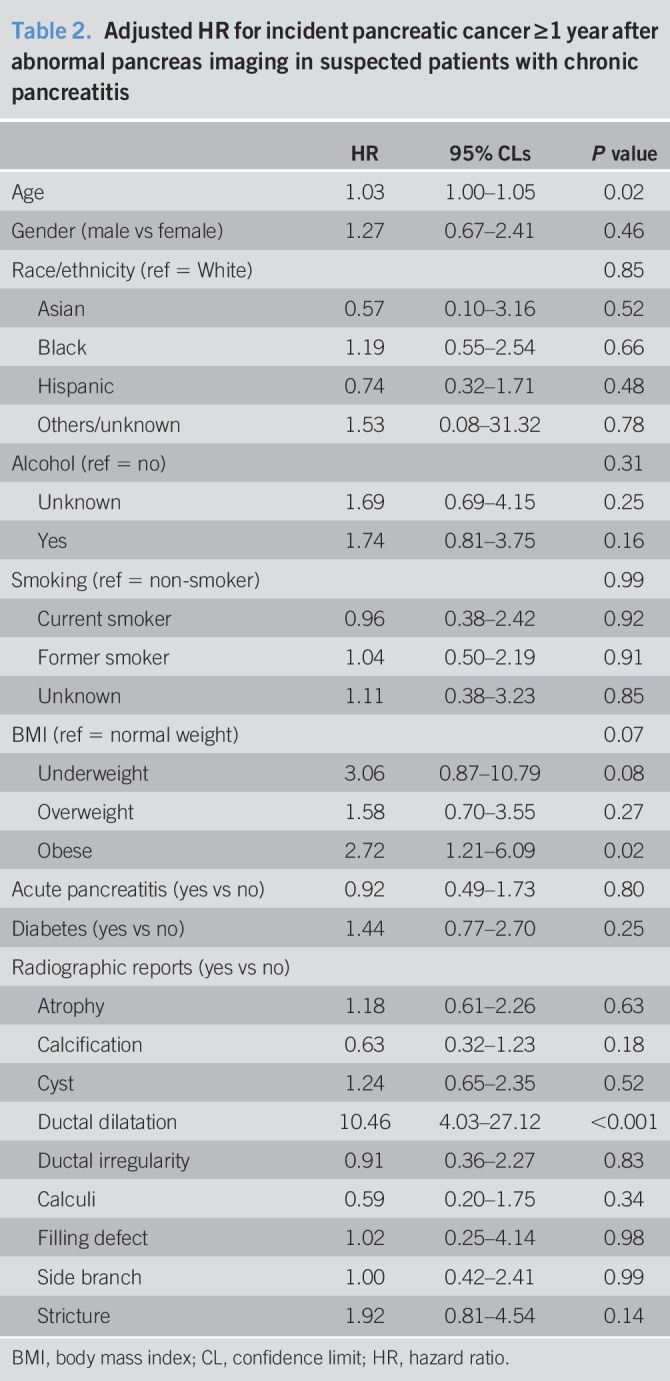
Adjusted HR for incident pancreatic cancer ≥1 year after abnormal pancreas imaging in suspected patients with chronic pancreatitis

**Figure 3.**
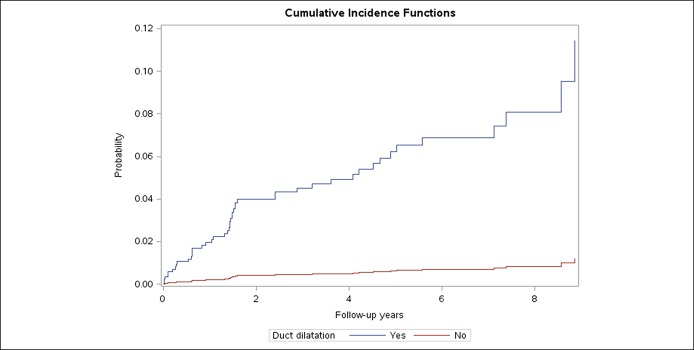
Cumulative incidence curve of pancreatic cancer ≥1 year after abnormal pancreas imaging in patients with suspected chronic pancreatitis by presence of ductal dilatation.

Duct dilatation was a salient feature of CP that was associated with the future risk of pancreatic cancer. The 2- and 5-year risks of pancreatic cancer in patients with CP with duct dilatation who survived 1 year without pancreatic cancer were 3.7% and 6.3%, respectively. By contrast, the 2- and 5-year risks of pancreatic cancer in CP patients without duct dilation were significantly lower: 0.6% and 0.6%, respectively (*P* < 0.001) (Figure 3). Although incidence also varied by the presence of calcification (2-year risk of 1.7% and 5-year risk of 3.2% in persons with calcification and 2-year risk of 2.3% and 5-year risk of 3.5% in persons without calcification), the differences were not statistically significant (*P* = 0.31).

The incidence rates of pancreatic cancer per 100,000 person years by sex and age group are presented in Supplementary Digital Content 1 (see Table S1, http://links.lww.com/CTG/A222). The standardized rate ratio of pancreatic cancer is presented in Supplementary Digital Content 1 (see Table S2, http://links.lww.com/CTG/A222). The incidence rate of pancreatic cancer in patients with CP was 12 times higher (incidence rate ratio 12, 95% CL: 8.8–16) than in the referent population, without adjustment for BMI.

## DISCUSSION

A major challenge in improving survival for patients that develop pancreatic cancer has been the lack of an effective strategy for early detection. In this community-based retrospective cohort study, patients with suspected CP were at substantially increased risk of developing pancreatic cancer compared with the age- and sex-matched reference populations. Patients with specific duct abnormalities who did not experience pancreatic cancer within 1 year were still at a >10-fold increased risk of developing subsequent malignancy as compared to patients with CP without duct dilatation. Moreover, we found that obese patients with CP experienced a 2.7-fold increased risk of pancreatic cancer as compared to patients with CP of normal weight. Periodic surveillance of these patients may help lead to early detection and treatment of pancreatic cancer.

We observed a 12-fold increased risk of pancreatic cancer in suspected patients with CP (see Table S2, Supplementary Digital Content 1, http://links.lww.com/CTG/A222) as compared to the referent population. Consistent with this, a recent meta-analysis of case-control and cohort studies estimated a relative risk of pancreatic cancer of 16.16 (95% confidence interval: 12.59–20.73) due to CP, with a 2-year lag period after CP diagnosis (2). Previous studies that have examined the risk of pancreatic cancer with no specific lag period showed a marked variation in relative risk estimates ranging from 1.5 to 28 based on the proximity in timing to the diagnosis of CP. The present study population was defined in a different manner compared with previous cohort studies that have defined CP based on either the ICD diagnosis codes only from national health registries (4,12,13) or clinical evaluation from the local disease registries (2,14–17). This distinction is important because our definitions incorporated both physician diagnosis of CP and pancreatic abnormality on imaging. By selecting patients with least 1 positive CP radiographic feature, we may have enriched the CP population to those with progression of pancreatic disease, potentially attributable to pancreatic cancer.

The 5-year incidence of pancreatic cancer (3.2%) in our study population of suspected patients with CP was even higher than the 10-year incidence of pancreatic cancer (1.8%) reported for the CP cohort populations investigated in the past (14). That the previous study estimated the risk of pancreatic cancer in persons with at least 2 years of follow-up, among younger individuals (median age: 40–50 years) over a recruitment period (1946–1989) when there were greater competing risks by death due to other causes, collectively contribute to the differences in pancreatic cancer risk estimates.

Duct dilatation was the most salient radiographic feature of CP that pointed to greatly elevated risk of pancreatic cancer, estimated at 6.3% in 5 years. The finding that pancreatic duct dilatation may be an early harbinger of pancreatic cancer has been previously demonstrated in a study of 27 patients with CP and 21 patients with pancreatic cancer (18). Conversely, another study of 427 patients undergoing endoscopic ultrasound, of whom 42 developed pancreatic cancer, did not substantiate this and rather attributed periductal hypoechoic sign as an indicator of pancreatic cancer (19). Our study of 1,766 suspected patients with CP, in whom 46 were diagnosed with pancreatic cancer, is the largest study, to date, establishing the relationship between CP radiographic features and the diagnosis of pancreatic cancer. Furthermore, we used the NLP-based methods for radiographic reports, thus developing a scalable algorithm for identifying CP features that foretells the future risk of pancreatic cancer.

Obesity was a risk factor for future development of pancreatic cancer in this cohort of suspected patients with CP. This is consistent with the trends in the general population, where obesity is associated with a 20%–50% increased risk of pancreatic cancer (20–22). Of many correlates of BMI, central obesity has shown a strong and consistent association with pancreatic cancer (20,23). Central obesity may promote tumor growth by way of causing inflammation in the pancreas. *In vivo* experiments also demonstrate that obese mice are prone to higher ductal cell replication (24) and development of pancreatic cancer (25).

There were several limitations to the present study. First, our definition of CP used a combination of diagnosis codes and imaging findings. Although this approach represents a clear advantage over reliance on the diagnosis codes alone, which has limited specificity (26), there remains a possibility of misclassification. Our imaging-based approach would likely fail to include patients with small-duct or “minimal change” CP (27) that may have been detected on endosonography or based on the results of pancreas function testing. Given that nearly a quarter of our study population underwent transabdominal ultrasound and less than 10% underwent MRI, accurate determination of duct diameter may not have been possible. Based on a manual review of the first abnormal pancreas radiograph of the patients with CP, only 42% of noncalcific CP showed a classic manifestation of CP, whereas 50% of patients had or developed acute pancreatitis without developing definite features of CP. Our manual review consisted of only 10% of the noncalcific patients with CP, and our algorithm has not been externally validated at other health systems. The accuracy of ICD coding we found is in line with the previous observation that ICD-coded diagnoses are limited in accurately identifying definite cases of CP (11). Among patients without a history of acute pancreatitis, timing of the disease onset regarding CP is very difficult to ascertain (28). This represents an underlying challenge in studying the natural history of CP because the ability to establish a diagnosis in the early stages of disease remains elusive (8). Given these limitations, our study population would be best described as persons with suspected CP rather than comprising solely of persons with definitive CP. In addition, there were only 46 patients with incident pancreatic cancer, thus limiting the power of the analysis to identify predictors of pancreatic cancer. Finally, a substantial portion of the study population were missing data on smoking (25%) and alcohol consumption (11%), which could have limited power and the accuracy of the relative risk estimates for smokers and alcohol consumers.

The strengths of the present study include the demonstration of an important role for state-of-the-art approaches to data acquisition such as NLP in helping to provide new insight into cancer prediction. Our methods were developed using open source tools and can be replicated across health systems with appropriate expertise in NLP. Previous applications of NLP on pancreatic disorders are few and have been limited to identifying existing pancreatic cysts (29,30) or IPMNs (31). These include NLP of surgical pathology texts to identify IPMNs beyond known IPMN cases in a manually curated registry (31) and NLP of any text-based reports in the medical records to identify pancreatic cysts or ductal dilatation (29,30), which had close concordance with manual review. Our study demonstrates that NLP can not only identify existing pancreatic abnormalities of immediate concern but also points to features that indicate high future risk of pancreatic cancer, thereby demonstrating utility for surveillance. In addition, our study had applied NLP to only reports of abdomen-focused imaging studies and thus was more selective than application of NLP to any reports in the medical records. Furthermore, our study cohort was drawn from a diverse community-based setting, thus our findings are generalizable to large health systems.

In summary, our study shows that in patients diagnosed with CP, obesity and duct dilatation on radiographic imaging point to persons with elevated longer-term risk of pancreatic cancer.

## CONFLICTS OF INTEREST

**Guarantor of the article:** Bechien U. Wu, MD, MPH.

**Specific author contributions:** B.U.W. conceived of the study design and acquired the data. S.J.P. and B.U.W. obtained funding. E.D. and B.U.W. performed the manual chart review. C.Y.J. and B.U.W. drafted the first draft of the manuscript. Q.C., W.Y., and J.C. conducted the statistical analyses. C.Y.J., E.D., S.J.P., D.Y., D.C., and B.U.W. contributed to interpretation of the results and the critical revision of the manuscript for important intellectual content.

**Financial support:** Research reported in this publication was supported by National Cancer Institute and National Institute of Diabetes and Digestive and Kidney Diseases of the National Institutes of Health under award number U01DK108314. S.J.P., C.Y.J., B.U.W. are supported in part by U01DK108314. D.C. is supported in part by U01DK108327, and D.Y. is supported in part by U01DK108306. The content is solely the responsibility of the authors and does not necessarily represent the official views of the National Institutes of Health.

**Potential competing interests:** None to report.

**Study Highlights**

**WHAT IS KNOWN**

✓ Pancreatic cancer occurs more often in patients with CP than in the general population. However, we lack guidelines for identifying which patients with CP should undergo surveillance for pancreatic cancer.

**WHAT IS NEW HERE**

✓ Among patients suspected for CP, ductal dilatation is a salient feature of future risk of pancreatic cancer.

**TRANSLATIONAL IMPACT**

✓ Active surveillance may be warranted in patients suspected with CP with ductal dilatation, given the elevated risk for pancreatic cancer.

## Supplementary Material

SUPPLEMENTARY MATERIAL
