# Supplementary material for: Identification of Individuals at Increased Risk for Pancreatic Cancer in a Community-Based Cohort of Patients With Suspected Chronic Pancreatitis
Source: Clin Transl Gastroenterol. 2020 Apr 4;11(4):e00147. doi: 10.14309/ctg.0000000000000147 (PMC7263650; doi:10.14309/ctg.0000000000000147)
Supplement: SUPPLEMENTARY MATERIAL [file ct9-11-e00147-s001.docx]

**Supporting Material**

The incidence rate of pancreatic cancer per 100,000 person-years by sex and age group are presented in **Table S1.** The overall incidence rate of pancreatic cancer after 1-year following the first positive pancreas image was 530 per 100,000 person-years, with highest incidence in the 61-70 year age category and slightly higher incidence in males vs. females. Of note, incidence of pancreatic cancer increased monotonically with increasing age in the general population, while incidence peaked at 61-70 years among suspected CP patients. Standardized to age and sex of the non-CP population, the incidence rate of pancreatic cancer in CP patients was 12 times higher (IRR 12, 95%CL 8.8, 16) (**Table S2**).

| **Table S1. Incidence of pancreatic cancer in suspected chronic pancreatitis patients vs. KPSC reference population during 2006-2016** | | | | | | | | | |
| --- | --- | --- | --- | --- | --- | --- | --- | --- | --- |
|  | **Incidence** | | | **Population size (person-years)** | | | **Incidence rate (per 100,000 person-years)** | | |
| **Age** | **Female** | **Male** | **Total** | **Female** | **Male** | **Total** | **Female** | **Male** | **Total** |
| **CP and surviving for one year^‡^** | | | | | | | | | |
| **≤40** |  |  |  | 411.2 | 429.7 | 840.9 |  |  |  |
| **41-50** | 2 | 3 | 5 | 573.4 | 714.4 | 1287.8 | 348.8 | 419.9 | 388.3 |
| **51-60** | 5 | 6 | 11 | 811.3 | 1168.3 | 1979.6 | 616.3 | 513.6 | 555.7 |
| **61-70** | 6 | 8 | 14 | 903.4 | 1141.9 | 2045.3 | 664.2 | 700.6 | 684.5 |
| **71-80** | 3 | 8 | 11 | 891.7 | 790.8 | 1682.5 | 336.4 | 1011.6 | 653.8 |
| **≥81** | 3 | 2 | 5 | 408.5 | 442.8 | 851.2 | 734.4 | 451.7 | 587.4 |
| **Total** | 19 | 27 | 46 | 3999.5 | 4687.8 | 8687.4 | 475.1 | 576.0 | 529.5 |
| **KPSC cohort (non-CP)** | | | | | | | | | |
| **≤40** | 81 | 74 | 155 | 10,460,922.7 | 10,229,409.6 | 20,690,332.3 | 0.8 | 0.7 | 0.7 |
| **41-50** | 166 | 173 | 339 | 2,831,851.9 | 2,652,282.0 | 5,484,133.9 | 5.9 | 6.5 | 6.2 |
| **51-60** | 489 | 589 | 1078 | 2,784,251.3 | 2,545,114.2 | 5,329,365.4 | 17.6 | 23.1 | 20.2 |
| **61-70** | 801 | 902 | 1703 | 1,968,210.8 | 1,727,217.3 | 3,695,428.2 | 40.7 | 52.2 | 46.1 |
| **71-80** | 825 | 769 | 1594 | 1,038,217.0 | 893,218.0 | 1,931,435.0 | 79.5 | 86.1 | 82.5 |
| **≥81** | 616 | 492 | 1108 | 533,503.6 | 362,657.7 | 896,161.4 | 115.5 | 135.7 | 123.6 |
| **Total** | 2978 | 2999 | 5977 | 19,616,957.5 | 18,409,898.8 | 38,026,856.2 | 15.2 | 16.3 | 15.7 |
| ^‡^Incidence of PC since 1-year after onset of CP among those who survived one year and didn’t developed PC. Age was calculated as of 1-year after onset of CP (N=1,766) | | | | | | | | | |

| **Table S2. Age and sex-standardized incidence rate ratio (IRR) of pancreatic cancer* for a cohort of suspected chronic pancreatitis patients vs. general population** | | | | |
| --- | --- | --- | --- | --- |
|  | **Pancreatic cancer** | | | |
|  | **IRR** | **95% Confidence Limits** | | **P-value** |
| **CP vs. general population** | 11.77 | 8.80 | 15.73 | <0.001 |
| **Age (Ref=40 or younger)** |  |  |  | <0.001 |
| 41-50 | 28.21 | 20.73 | 38.37 | <0.001 |
| 51-60 | 91.83 | 68.37 | 123.35 | <0.001 |
| 61-70 | 208.74 | 155.76 | 279.78 | <0.001 |
| 71-80 | 372.34 | 277.74 | 499.15 | <0.001 |
| >=81 | 561.89 | 418.34 | 754.61 | <0.001 |
| **Sex (male vs. female)** | 1.20 | 1.14 | 1.27 | <0.001 |

***** Pancreatic cancer after 1 year of abnormal pancreas imaging in suspected chronic pancreatitis among those who survived one year and didn’t developed pancreatic cancer. Age was calculated as of 1 year after abnormal pancreas imaging in suspected chronic pancreatitis patients.
